# Atherosclerotic plaque characteristics on quantitative coronary computed tomography angiography associated with ischemia on positron emission tomography in diabetic patients

**DOI:** 10.1007/s10554-022-02611-1

**Published:** 2022-05-09

**Authors:** Vasileios Kamperidis, Michiel A. de Graaf, Valtteri Uusitalo, Antti Saraste, Jurriën H. Kuneman, Inge J. van den Hoogen, Juhani Knuuti, Jeroen J. Bax

**Affiliations:** 1 Department of Cardiology, Heart Lung Centre, Leiden University Medical Centre, Albinusdreef 2, 2333 ZA, P.O. Box 9600, 2300 RC Leiden, The Netherlands; 2 Department of Cardiology, AHEPA University Hospital, Aristotle University, Thessaloniki, Greece; 3 Turku PET Centre, University of Turku, Turku, Finland; 4 Heart Center, Turku University Hospital, Turku, Finland; 5 Department Clinical Physiology, Nuclear Medicine and PET, University of Turku, Turku, Finland

**Keywords:** Diabetes mellitus, Coronary CTA, Coronary plaque quantification, PET, Myocardial ischemia

## Abstract

Patients with diabetes mellitus (DM) may show diffuse coronary artery atherosclerosis on coronary computed tomography angiography (CTA). The present study aimed at quantification of atherosclerotic plaque with CTA and its association with myocardial ischemia on positron emission tomography (PET) in DM patients. Of 922 symptomatic outpatients without previously known coronary artery disease who underwent CTA, 115 with DM (mean age 65 ± 8 years, 58% male) who had coronary atherosclerosis and underwent both quantified CTA (QCTA) and PET were included in the study. QCTA analysis was performed on a per-vessel basis and the most stenotic lesion of each vessel was considered. Myocardial ischemia on PET was based on absolute myocardial blood flow at stress ≤ 2.4 ml/g/min. Of the 345 vessels included in the analysis, 135 (39%) had flow-limiting stenosis and were characterized by having longer lesions, higher plaque volume, more extensive plaque burden and higher percentage of dense calcium (37 ± 22% vs 28 ± 22%, p = 0.001). On univariable analysis, QCTA parameters indicating the degree of stenosis, the plaque extent and composition were associated with presence of ischemia. The addition of the QCTA degree of stenosis parameters (*x*^2^ 36.45 vs 88.18, p < 0.001) and the QCTA plaque extent parameters (*x*^2^ 88.18 vs 97.44, p = 0.01) to a baseline model increased the association with ischemia. In DM patients, QCTA variables of vessel stenosis, plaque extent and composition are associated with ischemia on PET and characterize the hemodynamic significant atherosclerotic lesion.

## Introduction

Cardiovascular disease is a well-recognized complication of diabetes mellitus (DM) with detrimental impact on patient’s life [1]. Coronary computed tomography angiography (CTA) has repeatedly demonstrated diffuse coronary artery atherosclerosis in DM patients [2, 3]. DM is associated with high coronary plaque burden, more diseased coronary segments, more non-obstructive plaques compared to non-DM patients, and obstructive lesions in 30% of asymptomatic patients detected on coronary CTA [2–4]. Noteworthy, in DM patients, the degree of coronary stenosis detected on coronary CTA has no direct association with ischemia detected on single-photon emission computed tomography myocardial perfusion [5, 6]. However, in the general population, coronary CTA variables of atherosclerosis and beyond were predictive of ischemia detected with the same method [7, 8]. Thus, in DM patients, a more detailed evaluation of atherosclerosis (beyond the degree of coronary artery stenosis), and a better way of ischemia detection have to be applied. 

Recently, quantitative coronary CTA (QCTA) has evolved as a new technique that offers quantified, not only visual assessment, of the coronary plaque burden: the degree of obstruction, the plaque extent and composition can be automatically defined. QCTA has been validated and the provided variables have been associated with ischemia and cardiac events in the general population [7, 9–11]. Moreover, positron emission tomography (PET) has emerged as a non-invasive method that evaluates myocardial perfusion and quantification of myocardial blood flow, accurately identifying flow-limiting coronary lesions compared to invasive fractional flow reserve. [12, 13].

However, QCTA analysis to identify coronary plaque characteristics (degree of obstruction, the plaque extent and composition) causing myocardial blood flow reduction and ischemia on PET has never been applied in symptomatic DM patients. Therefore, the current analysis aims at identifying QCTA coronary plaque characteristics, beyond the degree of coronary stenosis, which are associated with myocardial ischemia on PET in DM patients.

## Methods

### Study population

In total, 922 consecutive symptomatic outpatients with suspected coronary artery disease and with intermediate pre-test likelihood for obstructive coronary artery disease underwent coronary CTA from 2007 till 2011 at the Turku PET Centre in Finland [10]. QCTA analysis was not feasible in 153 patients. Additionally, 261 had no atherosclerotic plaques on initial qualitative visual assessment. Of the 508 patients having any degree of atherosclerosis on coronary CTA, 295 underwent PET for ischemia detection based on clinical decision due to suspected obstructive CAD. Finally, 115 of these patients, who had DM and with feasible QCTA analysis and available PET were included in the current analysis. DM is defined as being on anti-diabetic treatment or having HbA1c ≥ 6.0% or plasma glucose > 6.0 mmol/l (diabetes and very high risk pre-diabetes) [14]. All the baseline demographic and clinical characteristics of the population and their survival data and the presence of a cardiac event defined as cardiac death, acute myocardial infarction, hospitalization for acute coronary syndrome and revascularization were prospectively collected and retrospectively analyzed from the departmental clinical database.

The study was performed according to the Declaration of Helsinki. The study was approved by the Ethics committee of the Hospital District of South-West Finland, and the need for patient written informed consent was waived.

### Coronary CTA image acquisition and quantitative analysis

All the patients included in the current analysis underwent a coronary CTA and a PET scan with a hybrid 64-row PET/CT scanner (GE Discovery VCT, General Electric Medical Systems, Waukesha, WI, USA). The coronary CTA scan was performed with collimation of 64 × 0.625 mm, gantry rotation time 350 ms, tube current 600–750 mA, voltage 100–120 kV according to patient size and prospective electrocardiographic triggering if feasible, according to the heart rate [10]. Before the coronary CTA acquisition, a bolus dose of 0–30 mg metoprolol was intravenously injected and 800 μg nitrate were sublingually administered. During the acquisition, 60–80 mL iomeprol (contrast agent of 400 mg iodine/mL) was infused at a rate of 4–4.5 mL/s, followed by saline flush.

**Fig. 1:**
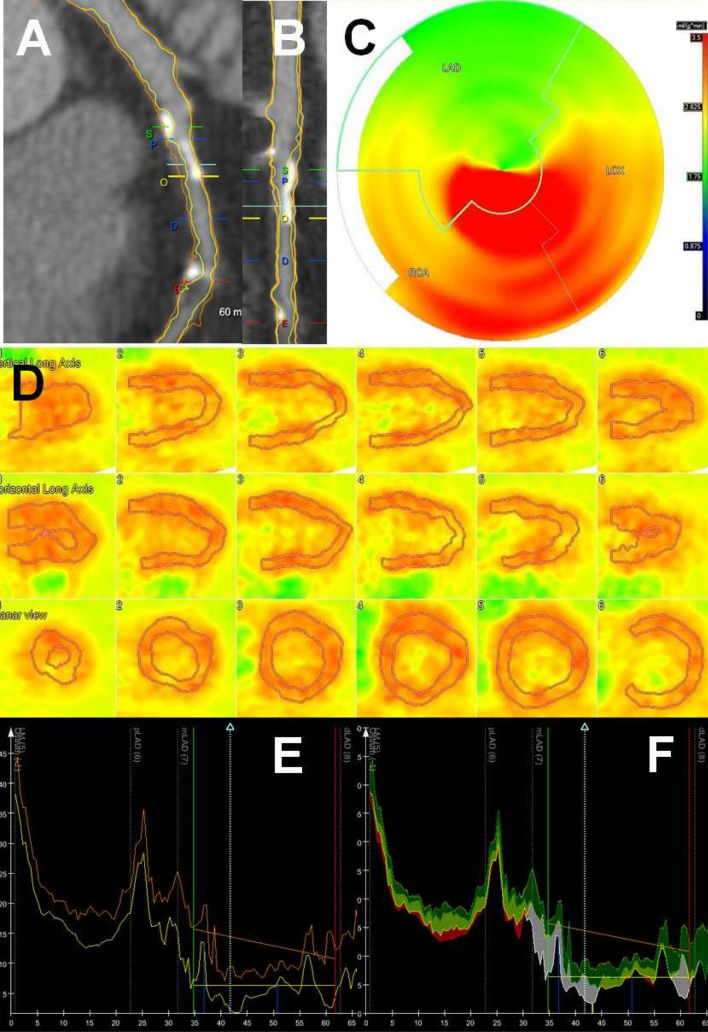
QCTA of the left anterior descending (LAD) coronary artery at the level of the most severe lesion of the vessel, causing ischemia of the anterior and anterior-septal wall of the left ventricle on PET in a male patient. Panels **A**, **B**, **E** and **F** QCTA reveals all the characteristics of a plaque in the mid LAD (mLAD) that are associated with ischemia: lumen area stenosis 73%, lumen area 1.07mm^2^, lesion length 14 mm, mean plaque burden 78.39%, calcium volume 25mm^3^. The yellow line is the lumen contour and the orange line is the vessel wall contour. The quantification of the most obstructive (O) lesion was done from proximal (P) to distal (D) border of the lesion, annotated by blue lines, and using as references the proximal region (S) and the distal region E, annotated by red lines. In Panel **F**, fibrotic tissue was labelled in dark green, fibro-fatty tissue in light green, dense calcium in white and necrotic core in red. Panels **C** and **D** PET at stress demonstrates the regional reduced perfusion of the anterior and anterior-septal wall due to ischemia. In the bulls-eye view (**C**), the green area indicates absolute blood flow of 1.75 ml/g/min which denotes ischemia

**Fig. 2:**
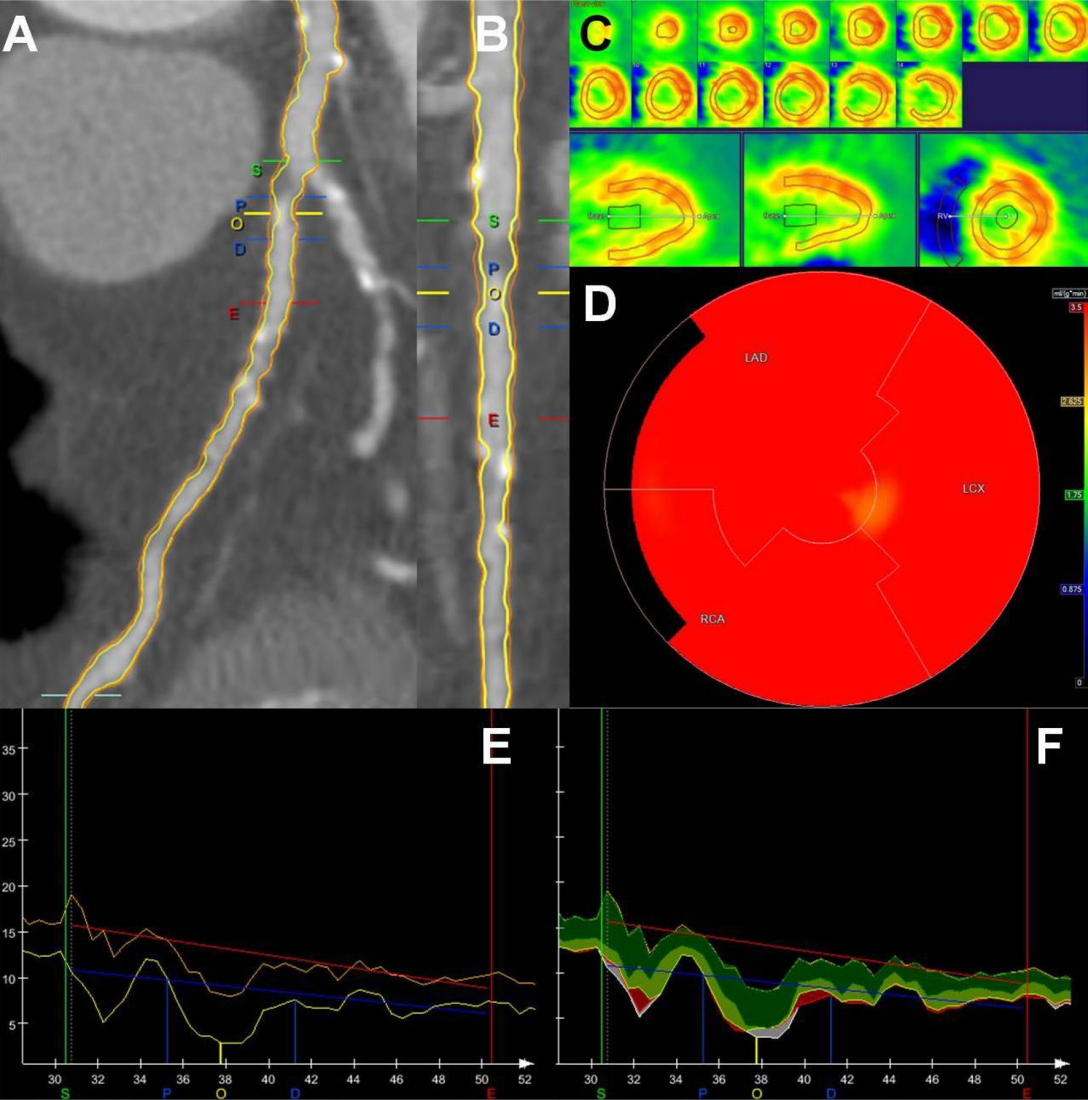
QCTA of the left anterior descending (LAD) coronary artery at the level of the most severe lesion of the vessel, not causing ischemia of the left ventricle on PET in a female patient. Panels **A**, **B**, **E** and **F** QCTA reveals all the characteristics of a plaque in the mid LAD (mLAD) that are associated with absence of ischemia: lumen area stenosis 61%, lumen area 3.16mm^2^, lesion length 6 mm, mean plaque burden 64%, calcium volume 1mm^3^. The yellow line is the lumen contour and the orange line is the vessel wall contour. The quantification of the most obstructive (O) lesion was done from proximal (P) to distal (D) border of the lesion, annotated by blue lines, and using as references the proximal region (S) and the distal region E, annotated by red lines. In Panel **F**, fibrotic tissue was labelled in dark green, fibro-fatty tissue in light green, dense calcium in white and necrotic core in red. Panels **C** and **D** PET at stress demonstrates normal perfusion of all the myocardial walls. In the bulls-eye view (D), the red area indicates absolute blood flow of 3.5 ml/g/min which denotes absence of ischemia

After the acquisition, the QCTA image analysis was performed using previously validated software (QAngio CT Research Edition, version 1.3.6; Medis Medical Imaging Systems, Leiden, the Netherlands) by an experienced reader (V.K. holds a level 3 accreditation by the Society of Cardiac Computed Tomography and is reading CT exams since 2013) blinded to the PET data [9, 10]. This software automatically extracted a 3-dimensional coronary tree and labelled each vessel and segment based on the American Heart Association 17-segment model [9]. The reader confirmed the multiplanar reformation of the vessels and the segmentation by revising the raw 3-dimensional data. The next step was the automatic quantification of all the atherosclerotic lesions of each vessel by the software based on the multiplanar reformation previously approved. For each lesion the analysis provided quantified data, as previously described [9], for the degree of lumen obstruction (lumen diameter, lumen area, lumen diameter stenosis and lumen area stenosis), for the plaque extent (lesion length, plaque volume, mean plaque burden and remodeling index) and for the plaque composition (dense calcium (≥ 350 HU) volume, fibrous tissue (131 to 350 HU) volume, fibro-fatty tissue (76 to 130 HU) volume and necrotic core (low-attenuation, -30 to 75 HU) volume). Consequently the reader picked the tightest lesion per vessel based on the degree of obstruction provided by the QCTA analysis. That was considered the potential lesion of each vessel causing ischemia of the corresponding myocardial territory. Thus, the QCTA analysis was performed on a-per-vessel basis. Figure 1 and Fig. 2 demonstrate two representative case examples.

On a per-patient-basis the software provided the number of obstructive and non-obstructive lesions, the number of proximal lesions and the number of calcified and mixed lesions.

### PET image acquisition and quantitative analysis

After coronary CTA image acquisition and first pass qualitative analysis, dynamic PET scan at rest and adenosine-induced stress was performed with the same scanner. The first step was the bolus injection of 900 to 1100 MBq ^15^O-labeled water (Radiowater Generator, Hidex Oy, Finland) over 15 s at an infusion rate of 10 mL/min. Then, a dynamic cardiac acquisition at rest was performed (14 × 5 s, 3 × 10 s, 3 × 20 s, and 4 × 30 s). After a 10-min interval of the first tracer injection, the adenosine infusion at a rate of 140 μg/kg/min started for the stress phase preparation and 2 min later another bolus of ^15^O-labeled water was given and the stress PET scan started. Adenosine infusion ended with the accomplishment of the stress phase.

After the acquisition, the quantitative image analysis was performed by an experienced reader (V.U.) blinded for the QCTA analysis using the previously validated Carimas™ software [15, 16]. On PET, 3 myocardial territories were defined based on the corresponding main coronary artery (1. left main and left anterior descending, 2. left circumflex, 3. right coronary artery). Absolute myocardial blood flow at stress ≤ 2.4 ml/g/min in any of the 3 vessel-based myocardial-territories was considered positive for myocardial ischemia, as has been previously defined [15, 17]. Overall, 345 (115 × 3) regions were analyzed and characterized as ischemic or not based on the absolute myocardial perfusion at stress.

### Statistical analysis

Continuous variables are presented as mean ± standard deviation if normally distributed and as median (interquartile range) if non-normally distributed. Categorical variables are expressed as frequencies (percentage). The cumulative cardiac-event-free survival rates were calculated based on Kaplan–Meier method and comparisons between groups were assessed by log-rank test.

Continuous QCTA variables are compared by using unpaired Student *t*-test based on the presence of corresponding ischemia on PET on a per-vessel-territory analysis. Univariable analysis was performed with binary logistic regression to detect QCTA variables associated with ischemia on PET on a per-vessel-territory analysis. A clinical multivariable model including age and gender was assessed for its association with ischemia on PET. Then, the incremental value of QCTA variables assessing the degree of obstruction, plaque extent and plaque composition were tested by adding them to the baseline clinical model on a stepwise approach, using binary logistic regression analysis. The odds ratio and 95% confidence interval were reported for uni- and multivariable analysis. The relative fit of the four tested models was calculated with the − 2 log likelihood. The four tested models were compared with chi-square (*x*^2^) test and c-statistics. Receiver operating characteristic analysis was used to assess the value of QCTA variables in predicting corresponding territory ischemia on PET, according to the Youden’s index (Sensitivity + Specificity-1), the sensitivity, the specificity and the area under the curve.

A p-value of < 0.05 was considered statistically significant. Statistical analysis was performed by SPSS (SPSS version 22, Inc., Chicago, IL, USA).

## Results

Table 1 describes the demographic characteristics of the 115 symptomatic DM patients (mean age 65 ± 8 years, 58% male). The coronary CTA characteristics of the population are provided in Table 1; the mean number of obstructive lesions was 2.61 ± 2.03, the mean number of proximal obstructive lesions was 1.30 ± 1.19 and the mean number of calcified plaques was 2.68 ± 2.21 per patient. The median calcium score was 293 (106; 727) AU (Table 1).

**Table 1:** Patient characteristics

	Diabetics (N = 115)
Clinical characteristics	
Age, years	65 ± 8
Male, n (%)	67 (58)
Blood glucose, mg/dl	124 ± 30
Glycated haemoglobin, %	6.55 ± 1.15
Family history, n (%)	49 (43)
Smoking, n (%)	13 (11)
Dyslipidemia, n (%)	95 (83)
Hypertension, n (%)	101 (88)
Chronic kidney disease: stage 1/2/3, n (%)	56(52)/ 45(42)/ 7(6)
Statin, n (%)	78 (78)
Aspirin, n (%)	81 (81)
Insulin, n (%)	17 (15)
CT characteristics	
Degree and extent of stenosis	
No of atherosclerotic plaques (< 30% stenosis)	4.17 ± 2.20
No of non-obstructive lesions (30–50% stenosis)	1.57 ± 1.16
No of obstructive lesions (> 50% stenosis)	2.61 ± 2.03
Location of obstructive lesions	
No of proximal lesions > 50% stenosis	1.30 ± 1.19
Composition and extent of stenosis	
No of partially calcified plaques (≥ 30% stenosis)	0.83 ± 0.99
No of calcified plaques (≥ 30% stenosis)	2.68 ± 2.21
No of non-calcified plaques (≥ 30% stenosis)	0.38 ± 0.66
Calcium score, AU	293 (106;727)

*AU* arbitary units, *CTA* computed tomography angiography, *No* number, *PET* positron emission tomography

**Fig. 3:**
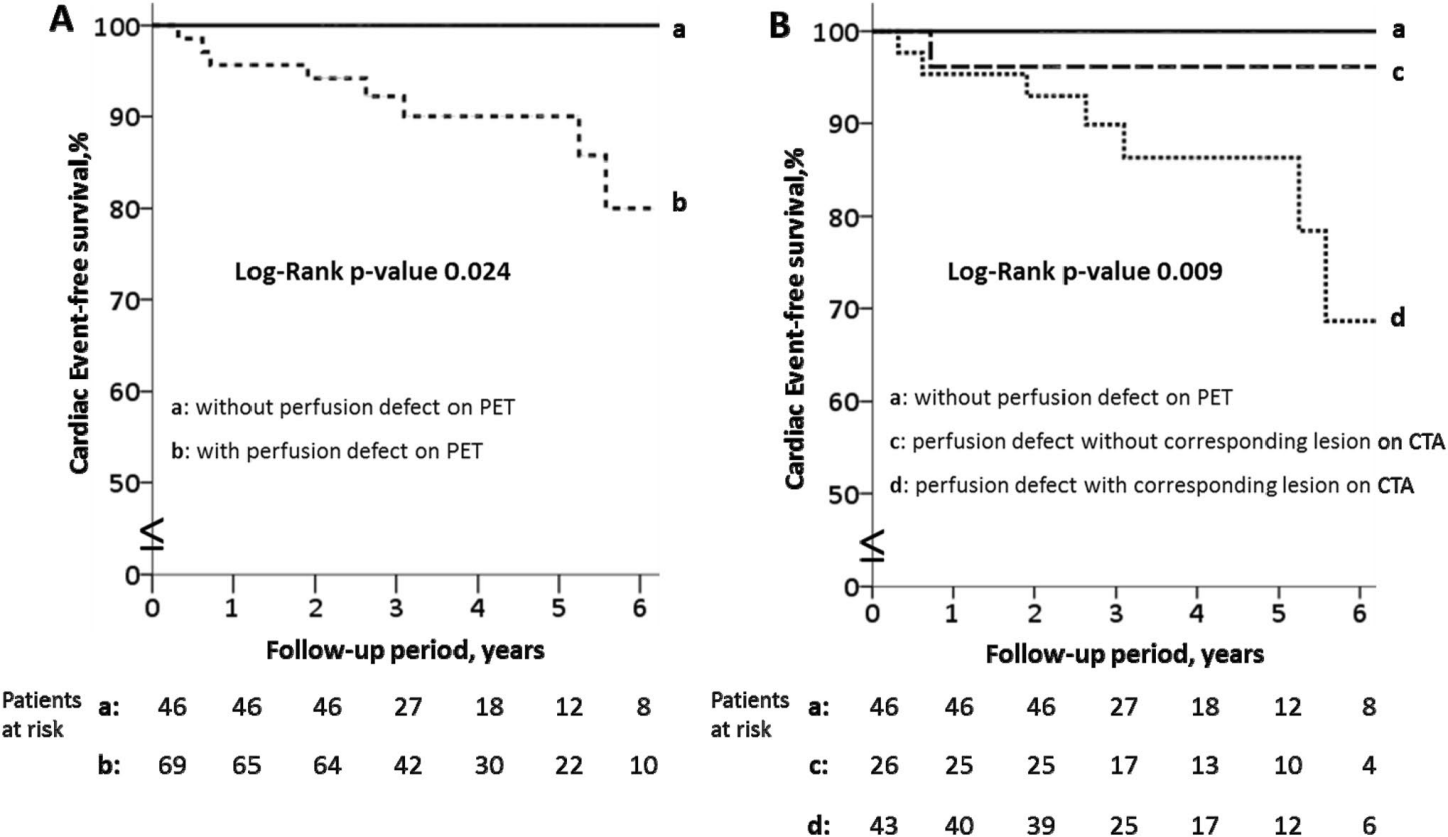
Kaplan–Meier analysis of cardiac-event-free survival according to: **Α** Perfusion defect on PET. **B** Perfusion defect on PET and atherosclerosis lesion on CTA

During a median follow-up period of 3.64 years (interquartile range 2.53—5.37 years) the patients without ischemia on PET had no events while those with ischemia had 8 cardiac events and cardiac-event-free survival rate 94%, 90% and 80% at 2-, 4- and 6-years respectively (rank-test p-value 0.024) (Fig. 3A). The patients with ischemia on PET and atherosclerotic lesion on the corresponding artery on CTA had worse cardiac-event-free survival rate (93%, 86% and 69% at 2-, 4- and 6-years respectively) compared with those without corresponding lesion on CTA and those without ischemia (rank-test p-value 0.009) (Fig. 3B).

Thus, the presence of an atherosclerotic lesion in the corresponding artery to the ischemic myocardial territory increases the chances of a cardiac event and makes it clinically relevant to focus on a per-vessel-territory analysis in order to identify QCTA atherosclerosis characteristics associated with ischemia on PET.

### QCTA characteristics and PET ischemia on a per-vessel analysis

In total 345 coronary arteries were included, corresponding to 345 myocardial territories with 135 of them (39%) having ischemia on PET. Vessels corresponding to ischemic territories on PET showed worse degree of obstruction as expressed by vessel’s lumen area, lumen area stenosis and diameter stenosis (Table 2). Moreover, these vessels had longer lesions, larger atherosclerotic plaque volume, more extensive plaque burden and showed more positive remodeling (Table 2). Additionally, vessels with flow-limiting lesions showed atherosclerotic plaques with larger volumes of calcium, necrotic core, fibrotic tissue and fibrous-fatty tissue (Table 2). Atherosclerotic plaques causing ischemia had higher percentage of dense calcium (37 ± 22% vs 28 ± 22%, p = 0.001) and lower percentage of fibrous-fatty tissue (9 ± 8% vs 14 ± 11%, p < 0.001) compared to vessels without flow-limiting lesions (Figs. 1, 2).

**Table 2:** QCTA characteristics of the most severe lesion per-vessel according to ischemia of the corresponding myocardial territory on PET

QCTA parameters	Total vessels (N = 345)	Vessel-territory ischemia on PET		P-value
		Yes (N = 135)	No (N = 210)	
Degree of obstruction parameters				
Lumen diameter, mm	1.32 ± 0.98	1.21 ± 0.80	1.39 ± 1.07	0.09
Lumen area, mm^2^	2.68 ± 2.78	2.11 ± 1.97	3.04 ± 1.15	0.002
Diameter stenosis, %	30.37 ± 24.35	40.82 ± 26.16	23.66 ± 20.54	< 0.001
Area stenosis, %	45.54 ± 31.82	58.11 ± 31.67	37.46 ± 29.25	< 0.001
Plaque extent parameters				
Lesion length, mm	9.64 ± 9.51	12.44 ± 10.51	7.84 ± 8.35	< 0.001
Plaque volume, mm^3^	71.47 ± 87.17	97.28 ± 106.51	54.87 ± 67.27	< 0.001
Mean plaque burden, %	39.77 ± 24.72	48.43 ± 23.87	34.21 ± 23.68	< 0.001
Remodeling index	0.69 ± 0.42	0.76 ± 0.38	0.64 ± 0.44	0.01
Plaque composition parameters				
Dense calcium volume, mm^3^	20.77 ± 36.66	31.18 ± 48.35	14.08 ± 24.45	< 0.001
Fibrotic tissue volume, mm^3^	25.54 ± 32.60	34.37 ± 37.80	19.86 ± 27.40	< 0.001
Fibrous-fatty volume, mm^3^	5.23 ± 6.80	6.31 ± 7.91	4.54 ± 5.89	0.02
Necrotic core volume, mm^3^	1.47 ± 2.56	1.82 ± 2.88	1.24 ± 2.30	0.04

*PET* positron emission tomography, *QCTA* quantitative computed tomography angiography

### QCTA parameters associated with ischemia on PET on a per-vessel-territory analysis

All the QCTA parameters expressing the degree of stenosis, the plaque extent and the plaque composition were associated with the presence of ischemia on univariable analysis (Table 3).

**Table 3:** QCTA parameters associated with corresponding ischemia on PET on a per-vessel-territory basis

	Univariable analysis		
	OR	95% CI	P-value
Degree of obstruction			
Lumen area stenosis, %	9.31	4.30–20.13	< 0.001
Lumen diameter, mm	0.83	0.66–1.03	0.090
Lumen area, mm^2^	0.87	0.80–0.96	0.003
Plaque extent			
Lesion length, mm	1.05	1.03–1.08	< 0.001
Plaque volume, mm^3^	1.01	1.00–1.01	< 0.001
Mean plaque burden, %	13.74	4.92–38.37	< 0.001
Remodeling index	1.94	1.14–3.30	0.015
Plaque composition			
Dense Calcium volume, mm^3^	1.02	1.01–1.02	< 0.001
Fibrotic tissue volume, mm^3^	1.02	1.01–1.02	< 0.001
Fibrous-Fatty volume, mm^3^	1.04	1.00–1.07	0.020
Necrotic core volume, mm^3^	1.09	1.00–1.19	0.049

*CI* confidence interval, *OR* odds ratio, *PET* positron emission tomography, *QCTA* quantitative computed tomography angiography

**Fig. 4:**
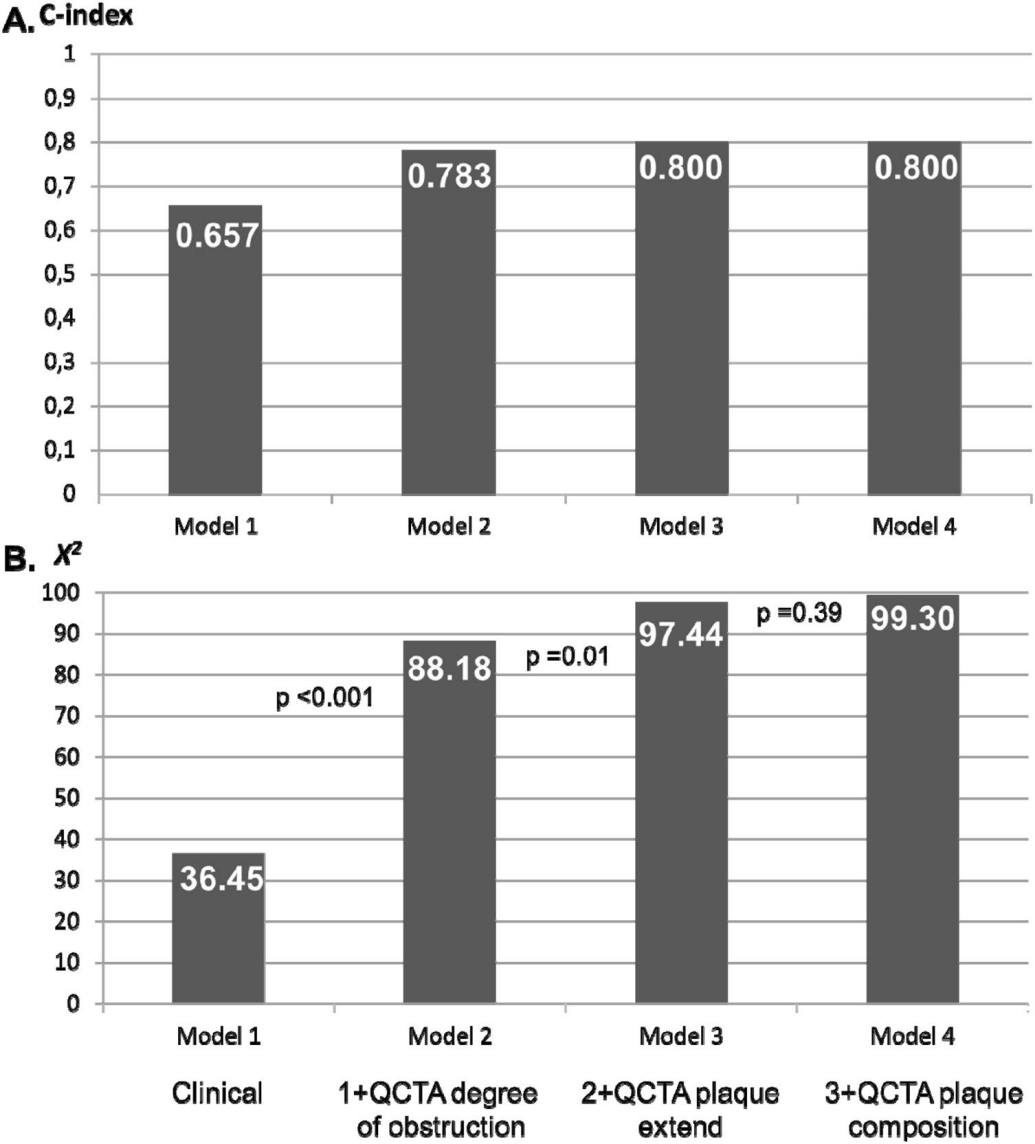
C-statistics and chi-square for comparing multivariable models of clinical and QCTA parameters identifying ischemia on PET, on a per-vessel-territory basis in diabetic patients. Bar graphs represent the (Panel **A**) c-index and (Panel **B**) *x*^2^ of each model. The addition of QCTA obstruction degree significantly increased the association of the clinical model with ischemia on PET. Furthermore, by adding the plaque extent, the association with ischemia incrementally increased. Finally, adding the plaque composition improved, but not significantly, the model’s association with ischemia on PET

Multivariable models were built to identify the additive incremental value of the QCTA plaque characteristics (Table 4). By adding the degree of stenosis parameters on QCTA, the baseline clinical model improved its association with ischemia (chi-square 36.45 vs 88.18, p < 0.001, c-statistics 0.783). At the next step, by adding the plaque extent QCTA parameters, the model association with ischemia further increased (chi-square 88.18 vs 97.44, p = 0.01, c-statistics 0.800). Finally, by adding the plaque composition, the association with ischemia did not increase further (chi-square 97.44 vs 99.30, p = 0.39, c-statistics 0.800) (Fig. 4).

**Table 4:** Multivariable models of clinical and QCTA associates of corresponding ischemia on PET on a per-vessel-territory basis

	Multivariable analysis				Model comparison		
	OR	95% CI	P-value*	-2 log likelihood	Chi-square	P-value†	C-index
Model 1 (clinical)				425.39	36.45	–	0.657
Age, years	1.01	0.98–1.04	0.63				
Male gender	4.27	2.59–7.06	< 0.001				
Model 2 (Model 1 + degree of obstruction)				373.66	88.18	< 0.001	0.783
Age, years	0.99	0.96–1.03	0.64				
Male gender	4.83	2.79–8.36	< 0.001				
Lumen area stenosis, %	11.14	4.91–25.28	< 0.001				
Lumen area, mm^2^	0.80	0.72–0.89	< 0.001				
Model 3 (Model 2 + plaque extent)				364.40	97.44	0.01	0.800
Age, years	0.99	0.96–1.03	0.57				
Male gender	5.22	2.98–9.16	< 0.001				
Lumen area stenosis, %	0.61	0.07–5.06	0.64				
Lumen area, mm^2^	0.72	0.62–0.84	< 0.001				
Lesion length, mm	1.03	1.00–1.07	0.058				
Mean plaque burden, %	31.94	2.03–502.12	0.014				
Model 4 (Model 3 + plaque composition)				362.54	99.30	0.39	0.800
Age, years	0.99	0.96–1.02	0.53				
Male gender	5.14	2.93–9.02	< 0.001				
Lumen area stenosis (%)	0.58	0.07–5.03	0.62				
Lumen area, mm^2^	0.71	0.60–0.83	< 0.001				
Lesion length, mm	1.00	0.95–1.06	0.91				
Mean plaque burden, %	37.34	2.19–636.22	0.012				
Calcium volume, mm^3^	1.00	0.99–1.01	0.40				
FibroFatty volume, mm^3^	1.03	0.97–1.10	0.27				

*CI* confidence interval, *OR* odds ratio, *PET* positron emission tomography, *QCTA* quantitative computed tomography angiography

The QCTA plaque characteristic of each category (degree of stenosis, plaque extent, plaque composition) that had the highest Youden index on receiver operating characteristic curve analysis to identify the best cut-off for association with territory ischemia on PET is: the lumen area stenosis with a cut-off value 42.50%, sensitivity 70%, specificity 58%, Youden index 28% (AUC 0.671, CI 0.615–0.728, p-value < 0.001), the mean plaque burden with a cut-off value 44.78%, sensitivity 69%, specificity 63%, Youden index 32% (AUC 0.686, CI 0.629–0.742, p-value < 0.001), the calcium volume with a cut-off value 9.47mm3, sensitivity 56%, specificity 73%, Youden index 29% (AUC 0.650, CI 0.592–0.709, p-value < 0.001).

## Discussion

The current study demonstrated that QCTA parameters assessing the stenosis degree (lumen area and area stenosis), the atherosclerotic plaque extent (lesion length, mean plaque burden) and the plaque composition (more calcified plaques) are associated with ischemia of the corresponding myocardial territory detected on PET in DM patients. The association with ischemia on PET was increased by adding the QCTA evaluated plaque extent parameters to the degree of lumen stenosis in DM patients.

### Atherosclerosis in diabetic patients

It is widely accepted that DM patients consist a special population with high prevalence of atherosclerosis and coronary artery disease. In an autopsy cohort of 2029 decedents, the diabetics had higher prevalence of coronary atherosclerosis and within the DM decedents without clinically known coronary artery disease 75% had high-grade atherosclerosis and 50% had multivessel coronary artery disease [18]. Coronary atherosclerosis in DM patients is not only an anatomical coincidence. Already from the Framingham study in 1979 it was well demonstrated that DM is a major predisposing factor to cardiovascular disease and it was not until recently that DM was considered coronary artery disease equivalent [19, 20]. Nonetheless, cardiovascular disease is the leading cause of morbidity and mortality in DM patients due to micro- and macrovascular disease; at every level of risk factors such as age, hypertension, hypercholesterolemia and smoking, the DM patients have at least twofold higher risk compared to non-DM individuals [21]. Thus, is of paramount importance in DM patients to detect coronary atherosclerosis in advance and this can trustworthy be done with the QCTA parameters of lumen stenosis and plaque extent that are indicative of ischemia on PET as demonstrated in the current analysis.

### Atherosclerosis CTA characteristics in diabetic patients

The extensive coronary atherosclerosis of the DM patients has been confirmed with CTA studies. Pundziute et al. analyzed a cohort of 215 symptomatic patients, 86 with DM, and demonstrated that the DM patients had more non-obstructive plaques and more coronary segments diseased on CTA, indicating more diffuse atherosclerosis [3]. These data have been confirmed by comparing CTA with intravascular ultrasound plaque extent and composition in 60 patients; the DM patients (n = 19) had more plaques and most were calcified on CTA and intravascular ultrasound [2]. These studies were based on visual assessment of the coronary plaques. A recent study that conducted QCTA analysis demonstrated that the plaque volume in DM patients evolves in a more pronounced way compared to non-DM subjects within 3.8 years scan interval indicating that the plaque volume on QCTA leads the atherosclerosis progression [22]. However, QCTA data are scarce for DM patients and the current analysis offers a deep insight in DM atherosclerosis burden highlighting the clinical value of plaque extent, plaque burden, which are in line with the aforementioned plaque volume, and lumen area besides the classical parameter of the stenosis degree.

The clinical value of CTA in asymptomatic DM patients has been demonstrated through long-term outcome studies. Kang et al. suggested that 31.6% of the DM patients had obstructive coronary artery disease on CTA and 6-year event free survival of 86%, which was significantly worse compared to those with non-obstructive disease or normal coronaries with survival 96% and 99% respectively. [23] In line, a meta-analysis of 6,225 DM patients concluded that CTA can safely rule out cardiovascular events in mid and long term follow-up and identify the high-risk patients with obstructive lesions [24]. Another study of asymptomatic DM patients performed QCTA analysis and identified the plaque characteristics that were associated with acute coronary syndrome in 9-years follow-up. The plaque volume, the low-density plaque content < 50 Hounsfields Units and the mild plaque calcification on QCTA were the parameters defining the lesion that was the culprit one 9-years later [25]. However, none of these studies either using CTA or QCTA characteristics studied symptomatic DM patients focusing on how to predict ischemia.

Thus, the question of how the QCTA in symptomatic DM patients correlates with myocardial ischemia at the time of the exam still waits to be elucidated. The current study resolved this query by suggesting that both stenosis degree and plaque extent define ischemia, in parallel with the aforementioned studies that correlated these CTA parameters with long-term events.

### Atherosclerosis QCTA characteristics associated with Ischemia detection in diabetic patients

This study is the first to associate QCTA characteristics with ischemia on PET in the corresponding myocardial territory in symptomatic DM patients.

Myocardial ischemia of symptomatic DM patients had been studied in an attempt to identify patients at need for further interventions. Giri et al. demonstrated that in the case of normal myocardial perfusion on stress single-photon emission computed tomography, the symptomatic DM patients had significantly worse cardiac survival compared to non-DM, indicating that this stress technique is not adequate to reassure the DM patients. [26] As a result a better stress technique for ischemia detection has to be applied. The resolution of the conundrum could be the use of ^15^O-labeled water dynamic PET with absolute quantitation of myocardial perfusion that is proven to have better diagnostic accuracy compared to scintigraphy in diagnosing ischemia against invasive fractional flow reserve which is the gold standard [27]. Thus, in the current study dynamic PET has been used for ischemia detection in the symptomatic DM patients.

However, in most symptomatic DM patients the first diagnostic test is CTA but not a stress test. According to PROMISE (Prospective Multicenter Imaging Study for Evaluation of Chest Pain) randomized trial, CTA diagnostic strategy is better for reducing adverse cardiovascular events compared to functional stress testing (adjusted hazard ratio 0.38, p = 0.01) [28]. Hence, it would be ideal to better identify the detailed CTA parameters that are predictive of myocardial ischemia. Diaz-Zamudio et al. studied 184 patients (15% with DM, 66% symptomatic) with QCTA and scintigraphy myocardial perfusion imaging and demonstrated that QCTA parameter of low-density non-calcified plaque burden was associated to corresponding territory ischemia on scintigraphy, independently of the stenosis degree [7]. However, besides the more tight stenosis, in the current analysis of DM patients it was not the low-density plaque burden, but the total plaque burden and the calcified plaques indicative of ischemia on PET, possibly reflecting the faster progression of atherosclerosis in DM patients. [3]

The invasive fractional flow reserve detects accurately ischemia that was predicted by the QCTA parameters of lesion length and plaque burden independently to stenosis severity in a study by Park et al., similarly to the current analysis, in a population including 21% DM patients [29]. Additionally, Kitabata et al. proved that lesions leading to 30–49% stenosis are associated significantly more with ischemia compared with lesions with < 30% stenosis [30]. All these results support that a severe stenotic lesion is not per se flow limiting while a non-severe should not be ignored.

Recently, the CTA derived fractional flow reserve (FFR-CT) has evolved as a new non-invasive method to identify the hemodynamically significant lesions detected on CTA in the general population and especially in DM patients, who often have it impaired [30]. Although the DM patients have more extensive atherosclerosis and more calcified vessels, causing blooming effect, the accuracy of FFR-CT was comparable between DM and non-DM patients [31]. In contrast to the current analysis, the abnormal FFR-CT ≤ 0.75, which is indicative of hemodynamic significant lesion, is associated with low-attenuation non-calcified plaques which is an established marker of high risk vulnerable plaque and has been predictive of adverse cardiac events in DM patients. [25, 32].

Therefore, this study is the first to select QCTA imaging and associate its parameters with corresponding ischemia on PET (best non-invasive method for ischemia detection) to further improve the diagnostic accuracy of CTA in symptomatic DM patients. Hence, the recognition of a lesion on QCTA, in a diabetic symptomatic patient, with tight lumen area stenosis, narrow lumen area, increased length and increased plaque burden is likely associated with ischemia and would guide the therapeutic choices accordingly taking into account not only the anatomy but the possible corresponding ischemia.

### Limitations

The current retrospective analysis of prospectively collected data is an observational study. The prognostic implications of QCTA parameters associated with ischemia on PET have to be demonstrated in prospective survival studies taking into account the pharmacological or interventional treatment applied based on QCTA and PET findings.

## Conclusion

In symptomatic DM patients undertaking CTA for diagnostic purposes, applying QCTA atherosclerosis analysis of the tightest per vessel lesion identified corresponding myocardial ischemia on PET. The QCTA parameters of area stenosis, lumen area, lesion length, plaque burden and plaque calcification are associated with ischemia on PET in patients with DM. The QCTA evaluated plaque extent parameters improved the prediction of ischemia beyond the degree of coronary lumen stenosis.

## Abbreviations

CTA: Computed tomography angiography
DM: Diabetes mellitus
PET: Positron emission tomography
QCTA: Quantitative coronary computed tomography angiography
